# Stabilizing Pd Catalysts for Liquid-Phase Hydrogenation of N‑Heterocyclic Hydrogen Carriers through Zeolite Encapsulation

**DOI:** 10.1021/acscatal.5c08818

**Published:** 2026-02-03

**Authors:** Sara Ahsan, Sirinada Chanthachaiwat, Alexander Kvit, Siddarth H. Krishna

**Affiliations:** † Department of Chemical and Biological Engineering, University of Wisconsin− Madison, 1415 Engineering Drive, Madison, Wisconsin 53706, United States; ‡ Department of Materials Science and Engineering, University of Wisconsin− Madison, 1509 University Avenue, Madison, Wisconsin 53706, United States

**Keywords:** zeolite encapsulation, metal catalyst, hydrogenation, sintering, hydrogen storage, catalyst stability

## Abstract

N-Heterocyclic aromatics can reversibly store H_2_ through (de)­hydrogenation over supported Pd catalysts, but metal nanoparticles often sinter during liquid-phase reactions. Here, we report that the encapsulation of Pd nanoparticles in large-pore zeolites stabilizes Pd catalysts during hydrogenation of *N*-methylindole (N-MID). Flow reactor studies combined with post-reaction characterizations show that Pd nanoparticles supported on SiO_2_ or Al_2_O_3_ sinter during hydrogenation of N-MID, while Pd/zeolites (particularly Pd/Beta) retain <2 nm particles, likely by suppressing the chelation and migration of Pd by N-MID. This work highlights the potential of zeolitic voids to suppress metal catalyst deactivation in liquid-phase reactions including H_2_ storage in chemical bonds.

## Graphical abstract

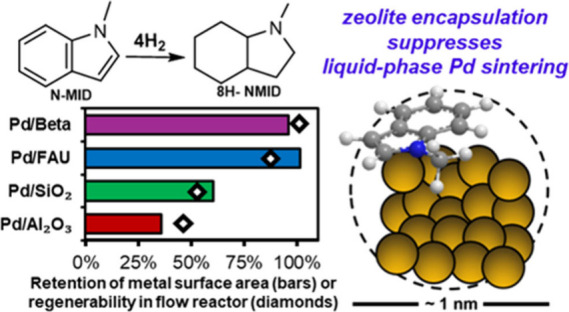

Stabilizing highly dispersed metal nanoparticles for liquid-phase catalytic reactions remains a major challenge for processes such as hydrogenations of organic molecules.^1,2^ Deactivation mechanisms such as coking and poisoning by organic species are often reversible upon high-temperature oxidative and reductive treatments; however, nanoparticle sintering often irreversibly decreases catalytic reactivity.^1^ In gas-phase reactions, high temperatures can drive sintering through mechanisms including thermally activated metal adatom migration (i.e., Ostwald ripening).^3,4^ In contrast, distinct deactivation mechanisms can prevail under liquid-phase reaction conditions: reactant and solvent molecules can chelate and mobilize surface metal atoms, causing sintering (from migration of surface-bound complexes) or leaching (from complete detachment of chelate complexes) at lower temperatures than in thermally activated processes.^5,6^ These challenges are particularly important in catalyst design for the (de)­hydrogenation of liquid hydrogen carriers (LHCs), which can store H_2_ reversibly in chemical bonds (Scheme 1).^7–9^ Aromatics such as toluene/methylcyclohexane have been studied as LHCs, but dehydrogenation steps require operation at >573 K, limiting energy efficiency.^10^ Incorporating nitrogen heteroatoms into aromatic rings, as in indoles^11–16^ and carbazoles,^17–21^ maintains similar H_2_ storage densities (∼6 wt %) but enables both hydrogenation and dehydrogenation steps to occur at <473 K.^22^ *N*-Methyl-indole (N-MID) is a particularly attractive N-containing LHC, as it is liquid across a wide temperature range (253–512 K)^14^ and achieves near-theoretical H_2_ storage capacities through selective hydrogenation and dehydrogenation.^23,24^ Hydrogenation of N-heterocycles is also important in the synthesis of some pharmaceuticals and fine chemicals.^25^

**Scheme 1:**
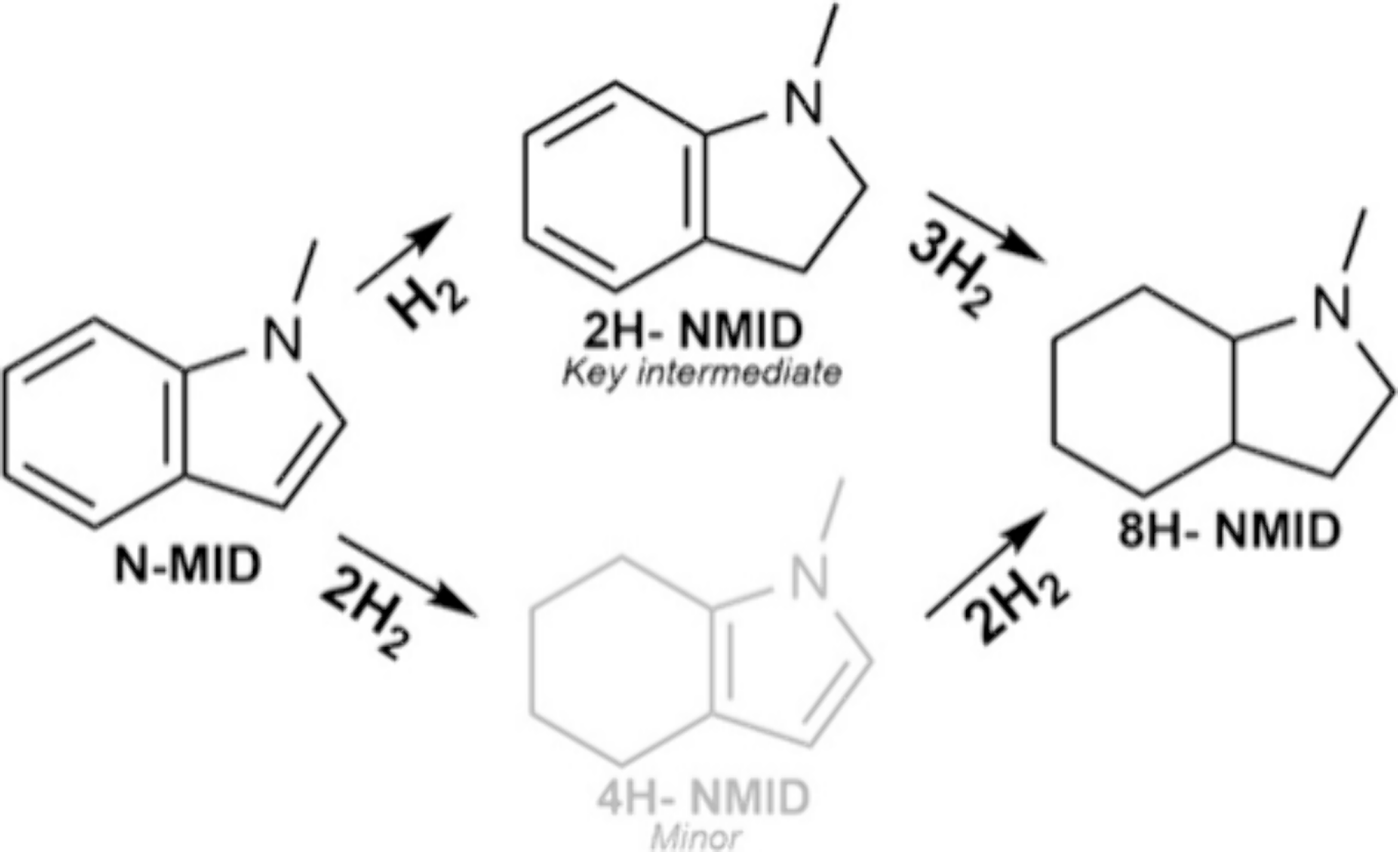
Reaction Network for N-MID Hydrogenation over Supported Pd Catalysts^a^

a Adapted with permission from Elsevier.^26^

We recently reported on the reactant-dependent stability of supported metal catalysts during hydrogenation of (methyl-)­indoles: while Pd and Ni supported on SiO_2_ or Al_2_O_3_ underwent coking with all studied N-LHCs that was reversible upon post-reaction regeneration treatments, the extent of (irreversible) sintering depended on the nature of both the LHC molecule and catalyst surface. Pd sintered in the presence of N-MID and 2-methyl-indoles (2-MID), but not with unsubstituted indole, suggesting that electron-donating methyl groups promote sintering. In contrast, Ni/SiO_2_ remained stable against sintering with indole and N-MID but sintered severely with 2-MID.^26^ Due to the chelating ability of pyrrole rings,^27^ we posited that sintering occurs via chelation-assisted migration of metal adatoms. These observations motivate advanced catalyst design strategies to protect metal nanoparticles against sintering in the liquid-phase hydrogenations of heterocyclic molecules.

Numerous strategies have been developed to stabilize supported metal nanoparticles against deactivation, including the strengthening of metal–support interactions,^28–32^ overcoating metal nanoparticles with porous oxides or carbon layers,^33–41^ and alloying.^42–45^ While these approaches can suppress sintering and coking to varying degrees, each has limitations: in overcoating strategies, the nanoscale morphology is difficult to control, leading to blocking of active sites and/or mass-transfer resistances that lower reactivity.^46^ Alternatively, metal nanoparticles can be encapsulated in the ordered nanopores of crystalline aluminosilicate zeolites to influence their structure and stability. Nanoparticle encapsulation in zeolites has been shown to suppress metal agglomeration in high-temperature gas-phase reactions.^47–52^ For example, confining noble metal clusters in zeolites improved stability against sintering at temperatures of ∼1073 K in CO_*x*_ conversion.^47^ Encapsulation of bimetallic alloys (Pt–Sn,^53^ Pt–Fe^54^) improved stability against coking in methylcyclohexane dehydrogenation.

Zeolite nanopores further influence species’ transport, adsorption, and/or reaction at confined metal surfaces through shape-selectivity effects.^55–60^ Iglesia and co-workers reported synthesis strategies to encapsulate various transition metal clusters in zeolite nanopores, demonstrating that such materials are size-selective in excluding bulky reactants and poisons from accessing intraporous metal clusters.^52,61,62^ Zeolite pores can also influence adsorbate binding and selectivity: Pt encapsulated in Faujasite (FAU) preferentially hydrogenated the C=O bond of cinnamaldehyde over the more reactive, but sterically hindered, C=C bond.^60^

In this work, we encapsulated metal nanoparticles in zeolites to suppress sintering during liquid-phase N-MID hydrogenation. We posit that zeolite nanopores of appropriate size should sterically suppress the migration of metal adatoms, which plausibly occurs via the formation of bulky metal-chelate complexes, while still permitting desired hydrogenation steps. To test this hypothesis, we synthesized Pd nanoparticles encapsulated in two commercial large-pore zeolites, FAU (Si/Al = 15) and Beta (Si/Al = 19), by incipient wetness impregnation (IWI) of palladium tetraamine nitrate. FAU contains 11.2 Å supercage voids connected by 7.4 Å apertures, while Beta contains 7.0 Å voids connected by 5.9 Å apertures;^63^ these dimensions are comparable to or larger than the critical diameter of indole (∼6.8 Å, based on similarly sized indane).^64^ During IWI, Pd precursors likely ion-exchange at zeolitic framework Al (Pd/Al < 0.06),^65^ subsequently forming Pd nanoparticles upon calcination (673 K, air) and reduction (523 K, H_2_) (material synthesis and characterization details are given in Section S1, SI). We compared these materials to Pd supported on two common oxide supports, Pd/SiO_2_ and Pd/Al_2_O_3_, to assess the effects of encapsulation on Pd sintering during N-MID hydrogenation.

Table S1, SI summarizes the physical and chemical properties of the synthesized 0.5 wt % Pd catalysts. The surface-area-weighted average particle diameters (*d*_STEM_)^66^ of Pd/FAU and Pd/Beta measured by scanning transmission electron microscopy (STEM) are 1.5 and 1.3 nm, respectively. These particle sizes are slightly larger than the pristine nanopore sizes of FAU and Beta, suggesting Pd nanoparticles may locally disrupt the framework as reported previously.^67,68^ CO chemisorption reported metal dispersions of 28% for Pd/FAU and 25% for Pd/Beta, which were used to normalize reaction rates per accessible Pd site. To assess associated Pd particle size distributions, we relied on STEM rather than CO chemisorption, as titration methods for encapsulated nanoparticles are complicated by issues including titrant accessibility to sites near pore walls.^69,70^

We performed shape-selective probe reactions, measuring hydrogenation rates of toluene (TOL) and 1,3,5-trisopropylbenzene (TIPB) to determine the extent of Pd encapsulation in zeolite pores, following reported approaches (Table 1; details are provided in Section S2.2, SI).^52,71^ The ratio of rates of TOL and TIPB hydrogenation (defined as χ, eq 1) was measured on Pd/SiO_2_ (where all Pd sites are accessible) and on Pd/zeolites. The ratio of χ values for Pd/zeolite and Pd/SiO_2_ (defined as φ, eq 2) enables estimating the fraction of Pd confined within zeolitic voids (eq S10, SI).

1 $$\chi =\frac{R_{Toluene}}{R_{TIPB}}$$

2 $$\varphi =\frac{\chi_{Pd/Zeolite}}{\chi_{Pd/SiO2}}$$

Pd/FAU and Pd/Beta displayed φ values of 7.1 and 5.7, respectively, indicating that >80% of Pd sites are encapsulated within zeolite pores in these materials.

**Table 1:** Assessing the Extent of Pd Encapsulation Using Shape-Selective Probe Reactions^a^

	Rate/mol (mol_surface.Pd_ * s)^−1^				
Catalyst	*R*_Tol_	*R*_TIPB_	χ (*R*_Tol_/*R*_TIPB_)	Φ (χ_Pd/Zeolite_ **/**χ_Pd/SiO2_)	Encapsulated Pd/%
Pd/FAU	0.81	0.022	36.8	7.1	86
Pd/Beta	3.55	0.12	29.6	5.7	82
Pd/SiO_2_	0.11	0.021	5.2	-	

a Conditions: 80 mM TOL or TIPB in dodecane (55 mL), 35 bar H_2_, 423 K. Catalyst masses: 0.51% Pd/FAU (TOL, 45 mg cat.; TIPB, 350 mg cat.), 0.52% Pd/Beta (TOL, 14 mg cat.; TIPB, 72 mg cat.), 0.58% Pd/SiO_2_ (TOL, 80 mg cat.; TIPB, 150 mg cat.).

We next studied the reactivity, time-on-stream (TOS) stability, and regenerability of supported Pd catalysts for the hydrogenation of 1 wt % N-MID (in dodecane as an inert solvent) in a continuous flow reactor at 373 K over Pd/FAU and Pd/Beta, compared to Pd/SiO_2_ and Pd/Al_2_O_3_ reported by us previously (Figure 1).^26^ Flow experiments were performed at low initial N-MID conversions (10–25%) to extract intrinsic reaction rates and stabilities, under which conditions the major product was typically 2H-NMID and the carbon balance was >95% (detailed yields are provided in Figure S4, SI). We excluded the influence of external and internal mass transport limitations on measured rates,^72,73^ described in Section S3.2, SI. Catalytic turnover numbers (mol_product_ mol_surface Pd_^–1^) were 1,000–4,300 for flow experiments. We characterized spent, regenerated catalysts via CO chemisorption following batch reactions with 2 wt % N-NMID at 423 K (Figure S7, SI) to understand the extent of sintering.^26^ Batch reactions displayed >90% selectivity toward desired hydrogenation products after correcting for N-MID adsorption to supports, ruling out significant side-reactions on Pd or zeolitic acid sites (Figure S8, SI).

**Figure 1:**
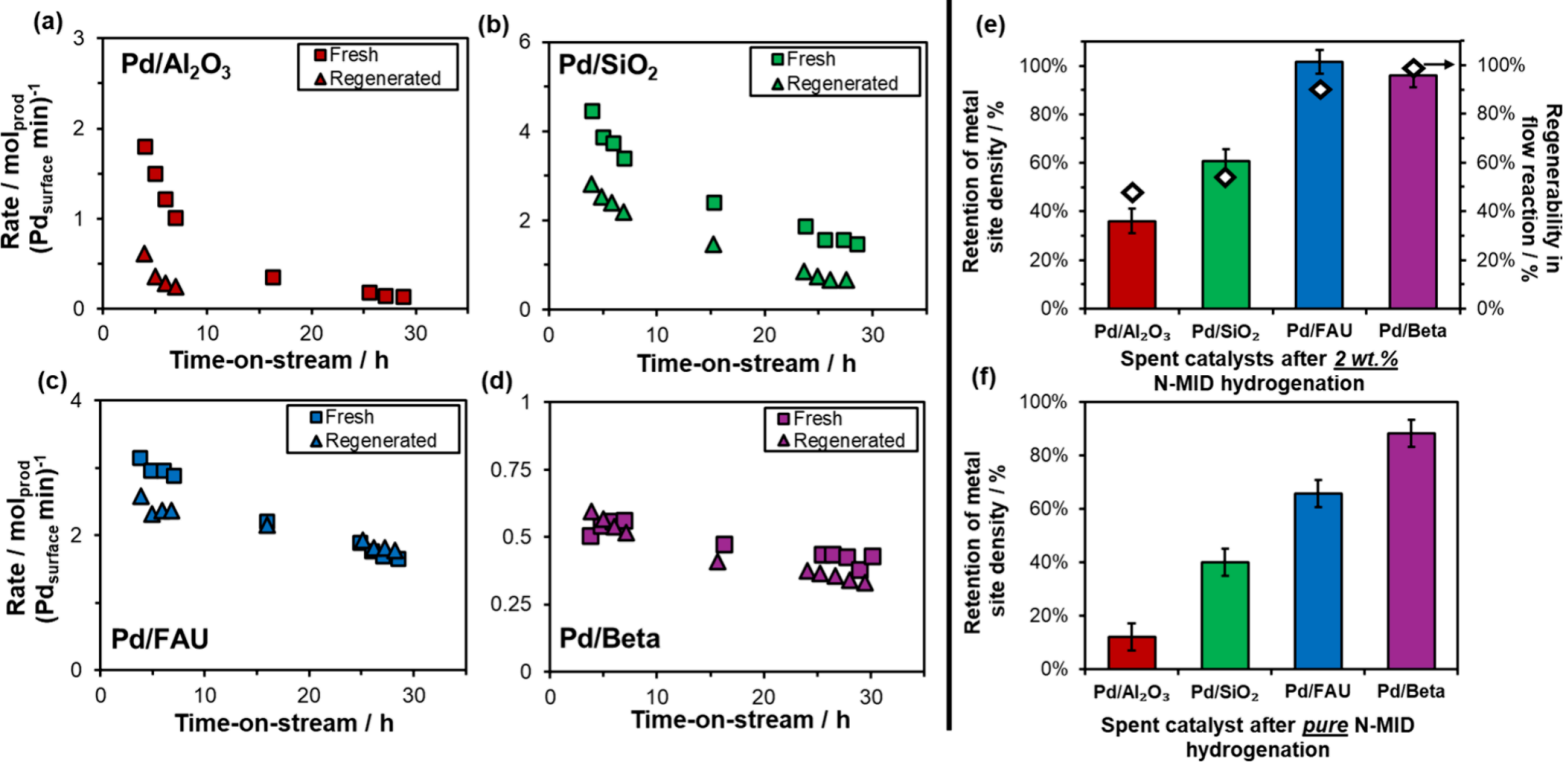
(a–d) Reaction rates versus time-on-stream (normalized per surface Pd on fresh catalysts) for hydrogenation of 1 wt % N-MID in a flow reactor over (a) 3.0 mg 0.65% Pd/Al_2_O_3_, (b) 3.0 mg 0.58% Pd/SiO_2_, (c) 10.1 mg 0.51% Pd/FAU, and (d) 25.0 mg 0.52% Pd/Beta [1.8 mL h^–1^ of 1 wt % N-MID in dodecane, 35 bar H_2_, 373 K]. Data over Pd/Al_2_O_3_ and Pd/SiO_2_ are reproduced from our prior work, with permission from Elsevier.^26^ Regeneration: calcination (673 K, air) and then reduction (523 K, H_2_). (e) Retention of Pd sites determined via CO chemisorption (bars) on spent, regenerated catalysts after hydrogenation of 2 wt % NMID in a batch reactor, versus the extent of catalyst regenerability in flow reactor measurements (diamonds). (f) Retention of Pd sites on spent, regenerated catalysts after batch hydrogenation of pure NMID. Further details are provided in Sections S3 and S4, SI.

During hydrogenation of 1 wt % N-MID, Pd/FAU and Pd/Beta were regenerable following deactivation with TOS, in sharp contrast to conventional supports Pd/SiO_2_ and Pd/Al_2_O_3_ (Figure 1). Over Pd/FAU, reaction rates decreased from 3.0 to 1.6 min^–1^ (Figure 1c) over 30 h of TOS. The majority (87%) of the initial reactivity is recovered after calcination and reduction, indicating that deactivation is predominantly due to coking (a small fraction of Pd located outside of zeolitic voids may sinter, see Table 1). In an analogous batch experiment, Pd/FAU retained its initial dispersion according to CO chemisorption (Figure 1e), in quantitative agreement with the reversibility of catalyst deactivation observed in the flow reactor. Pd/Beta (Figure 1d) deactivates more mildly over 30 h of TOS (rates decrease from 0.5 to 0.4 min^–1^). Reactivity is fully recovered after calcination and reduction treatments, and Pd/Beta retained its initial dispersion according to CO chemisorption (Figure 1e). In sharp contrast, Pd/SiO_2_ and Pd/Al_2_O_3_ deactivate more severely over 30 h of TOS (e.g., rates on Pd/SiO_2_ decrease from 4.4 to 1.4 min^–1^).^26^ Regeneration only recovered ∼50% of the initial reactivity and 36–60% of the initial Pd sites (Figure 1e) measured via CO chemisorption on spent, regenerated catalysts, consistent with sintering observed by us previously in STEM images.^26^ Pd/SiO_2_ and Pd/Al_2_O_3_ also exhibited a greater extent of coking (7.5–9.3 wt %; Table S4, SI) than Pd/zeolites (∼3 wt %); zeolite confinement may mitigate coke formation by restricting growth of bulky carbonaceous species.^74^

We next investigated Pd sintering resistance in the hydrogenation of pure N-MID at 423 K in a batch reactor, which maximizes H_2_ storage density in practical LHC technologies and likely exacerbates reactant-induced sintering mechanisms. N-MID conversions (8–76%) and turnover numbers (>2,100) are provided in Table S3 and Figure S7, SI. Pd/SiO_2_ retained only 29% of initial Pd sites following hydrogenation of pure N-MID (Figure 1f), consistent with a 76% increase in the surface-averaged Pd particle size from STEM (Figure 2c,d). In addition to sintering, 25% of Pd leached from Pd/SiO_2_ (Table S5, SI), consistent with chelation by N-heterocyclic reactants. The greater severity of sintering with pure N-MID compared to 1–2 wt % NMID shows that sintering depends on N-LHC concentration.

**Figure 2:**
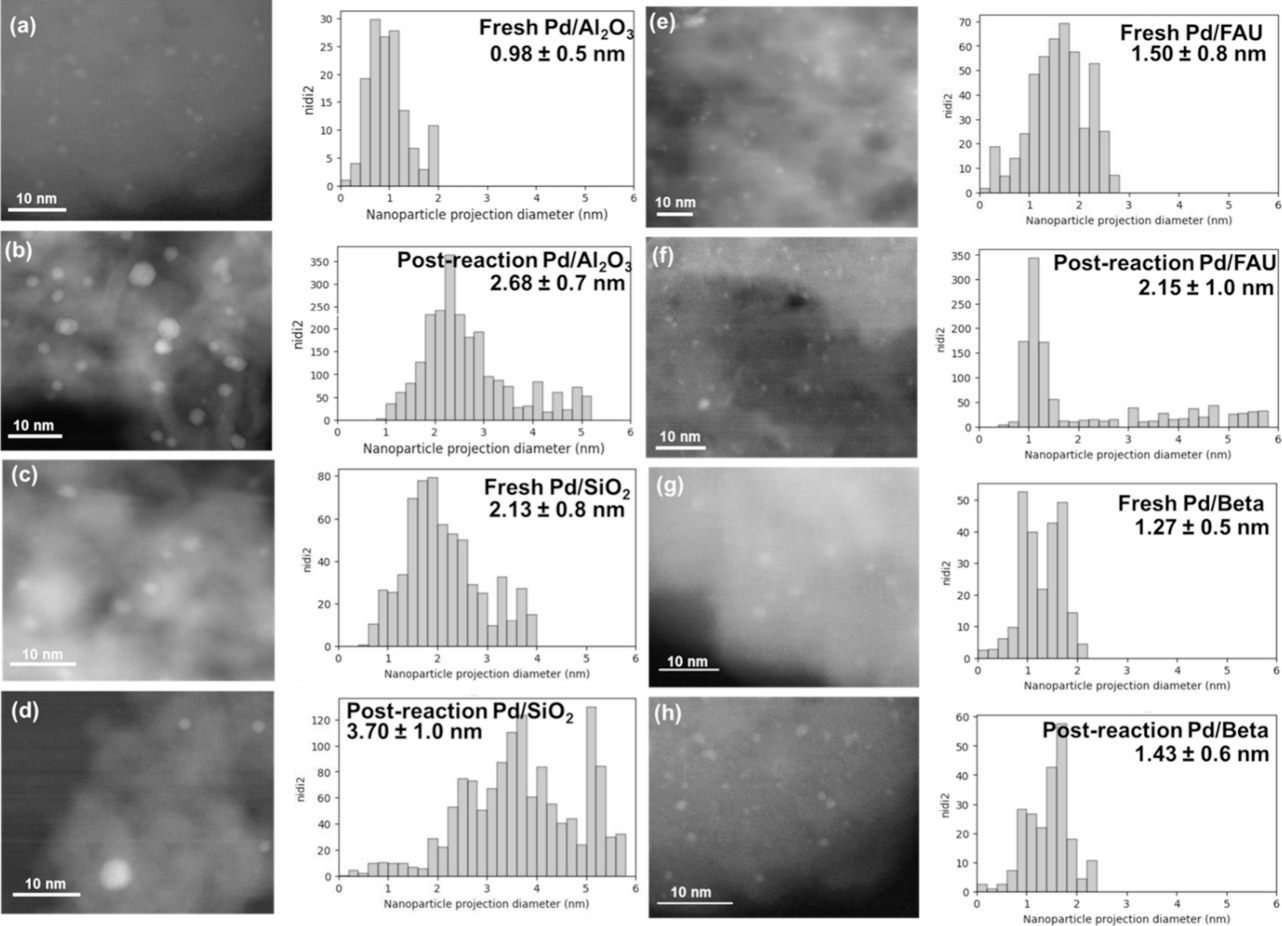
Representative STEM images and surface-averaged Pd particle size distributions of fresh (a) Pd/Al_2_O_3_, (c) Pd/SiO_2_, (e) Pd/FAU, and (g) Pd/Beta; and of spent, regenerated (b) Pd/Al_2_O_3_, (d) Pd/SiO_2_, (f) Pd/FAU, and (h) Pd/Beta following batch hydrogenation of pure N-MID (reaction results are provided in Figure S7b, SI). A small number of larger nanoparticles were observed on Pd/Beta; see Figure S2, SI.

A similar extent of sintering with N-MID was observed in an inert gas environment at 423 K (Figure S9, SI), showing that N-MID induces Pd migration, even in the absence of hydrogenation catalysis. The pure fully hydrogenated product, 8H-NMID, also induces sintering of Pd/SiO_2_ (at a higher temperature of 453 K relevant to dehydrogenation, but under 35 bar of H_2_ where 8H-NMID is unreactive), resulting in only 46% retention of Pd sites (Figure S10, SI). Similarly to Pd/SiO_2_, Pd/Al_2_O_3_ sinters severely during pure N-MID hydrogenation, retaining only 13% of its initial site density (Figure 1f), corroborated by STEM imaging (Figure 2a,b). Pd/Al_2_O_3_ also sinters in the presence of 8H-NMID (453 K, under H_2_; Figure S10, SI). Thus, both saturated and unsaturated N-heterocyclic rings induce severe sintering of conventional supported Pd catalysts, highlighting the challenge of stabilizing catalysts for N-heterocycle (de)­hydrogenations.

In sharp contrast, Pd/Beta retained 88% of its site density in the hydrogenation of pure N-MID (while performing 62,800 turnovers; Table S3, SI), consistent with no significant change in Pd particle size (Figure 2g,h), showing that the smaller voids of Beta greatly suppress reaction-induced sintering. Pd/Beta was also resistant to sintering with 8H-NMID in the presence of H_2_ (Figure S10, SI). Pd/FAU retained 66% of its initial Pd site density following pure N-MID hydrogenation (Figure 1f), suggesting that the larger 1.1 nm supercage voids of FAU provide partial stabilization against sintering but still permit some reaction-induced Pd migration and sintering (consistent with STEM imaging, Figure 2e,f); we cannot rule out that the small fraction of Pd located outside of nanoporous voids (Table 1) influences this comparison between Pd/FAU and Pd/Beta. Batch reactor studies of the reverse reaction, 8H-NMID dehydrogenation, under dilute conditions (453 K, 1 wt % 8H-NMID in dodecane, under inert Ar) show that Pd/Beta is also resistant to sintering and selective toward desired dehydrogenation products (4H-NMID, NMID) at the studied conditions (Figures S12, S13; SI); detailed catalyst reactivity and stability assessments in 8H-NMID dehydrogenation are reserved for future work.

Table 2 reports further analyses of the initial rate and deactivation behavior in continuous flow reactions of 1 wt % NMID hydrogenation from Figure 1. To assess whether lower initial rates on Pd/Beta (9 times lower than Pd/SiO_2_) are due to intracrystalline transport limitations,^73^ we computed the Weisz–Prater number (*N*_wp_) (Section S3.2, SI), which suggests that transport effects do not significantly hinder rates of N-MID hydrogenation in Pd/Beta. Nonetheless, the smaller voids of Beta relative to FAU and conventional oxide supports may alter N-MID adsorption or hydrogenation transition states through steric, electronic, or other effects to lower the hydrogenation rate.^75^ While structure-sensitivity effects are well-documented in Pd-catalyzed hydrogenation reactions in general^76^ and N-LHC (de)­hydrogenations in particular,^21^ this work focuses on the influence of encapsulation on sintering stability; detailed investigation of structure-sensitivity effects of zeolite-encapsulated Pd is reserved for future work.

**Table 2:** Initial Reaction Rates and Site-Loss Turnover Numbers (SLT) during Hydrogenation of 1 wt.% N-MID at 373 K, Extracted from Flow Data on Fresh Catalysts in Figure 1 ^a^

Catalyst	Rate/min^–1^	SLT/(mol_product_) (mol _sites lost_)^−1^	*k*_deactivation_/10^–4^ min^–1^
Pd/Beta	0.58 ± 0.09	2,270 ± 500	1.9 ± 0.3
Pd/FAU	3.3 ± 0.13	8,110 ± 450	4.2 ± 0.1
Pd/SiO_2_ ^a^	5.3 ± 0.42	6,470 ± 1,030	7.1 ± 0.3
Pd/Al_2_O_3_ ^a^	2.7 ± 0.41	1,150 ± 250	17 ± 0.8

a Deactivation model fits are provided in Figure S5, SI.

b Data reproduced from our previous work with permission from Elsevier.^26^

To compare the TOS stability in continuous flow N-MID hydrogenation across catalysts, including (reversible) coking and (irreversible) sintering, we calculated two different catalyst stability metrics (Table 2): the first-order deactivation rate constant (*k*_d_; eq S7, SI) and the site-loss turnover number (SLT; eq S8, SI). Mathematically, the SLT is the inverse of the cumulative site-loss selectivity discussed by Bhan and co-workers^77^ and estimates the number of turnovers each metal site would perform before deactivation, assuming all sites deactivate similarly. The *k*_d_ values decreased in the following order: Pd/Al_2_O_3_ > Pd/SiO_2_ > Pd/FAU > Pd/Beta. The higher *k*_d_ values of Pd/Al_2_O_3_ and Pd/SiO_2_ likely reflect the fact that these catalysts both coke and sinter, in contrast to Pd/zeolites, which do not sinter under these conditions. In contrast, SLT values (for which larger values indicate a greater number of turnovers relative to catalyst deactivation events) follow the trend Pd/Al_2_O_3_ < Pd/Beta < Pd/SiO_2_ < Pd/FAU; SLT measurements penalize Pd/Beta for having a lower N-MID hydrogenation rate. Still, the near-complete regenerability of Pd/Beta in the hydrogenation of pure N-MID offers a key advantage in catalyst stability over the other Pd catalysts studied here.

Summarizing, we have shown that Pd encapsulation within larger-pore zeolites provides a strategy to suppress sintering during the hydrogenation of N-heterocyclic LHCs. While both Pd/FAU and Pd/Beta resist sintering under dilute reactant conditions, the more constrained pore environment of Beta offers superior Pd stabilization in the hydrogenation of pure N-MID, albeit at the expense of lower reactivity. Although this work provides strong evidence for reactant-induced sintering of Pd by N-MID, additional computational modeling and experimental characterizations are needed to understand the detailed molecular mechanisms of chelation and metal migration. Beyond advancing our fundamental understanding of structure–stability relationships through nanoparticle encapsulation in zeolites for N-MID hydrogenation, this work offers guidance for the design of stable catalysts in liquid-phase organic reactions where reactant-induced metal sintering may occur, such as in conversion of O- and N-containing organic molecules relevant to LHC (de)­hydrogenation,^26,78^ biomass upgrading,^79–81^ and in fine chemical^82^ and pharmaceutical syntheses.^83^ We anticipate that our approach can be extended to other reaction systems by tuning encapsulated site pore environments to permit the desired adsorption and reaction steps while suppressing the formation of bulkier chelate complexes of the reacting organic species with catalytic metal centers.

## Supplementary Material



## Data Availability

Data are provided in a publicly available Zenodo repository.^84^

## Notes

Experimental procedures, reaction analyses, and catalyst characterization (PDF)
