# Supplementary material for: Stabilizing Pd Catalysts for Liquid-Phase Hydrogenation of N‑Heterocyclic Hydrogen Carriers through Zeolite Encapsulation
Source: ACS Catal. 2026 Feb 3;16(4):3097–104. doi: 10.1021/acscatal.5c08818 (PMC12930350; doi:10.1021/acscatal.5c08818)
Supplement: Supplementary file 1 [file cs5c08818_si_001.pdf]

ACS Catalysis

Supplemental Information:

Stabilizing Pd catalysts for liquid-phase hydrogenation of N-heterocyclic hydrogen carriers through zeolite encapsulation

Sara Ahsan[1], Sirinada Chanthachaiwat[1], Alexander Kvit[2], Siddarth H. Krishna[1]*

[1]Department of Chemical and Biological Engineering, University of Wisconsin- Madison, 1415 Engineering Drive, Madison, WI 53706, USA

[2]Department of Materials Science and Engineering, University of Wisconsin- Madison, 1509 University Avenue, Madison, WI 53706, USA

*Corresponding author: krishna3@wisc.edu

S1. Materials and Methods.

S1.1 Materials

N-methylindole (99%, Oakwood Chemicals), dodecane (99%, TCI Chemicals), $Pd(NH_3)_4(NO_3)_2$ (10 wt.% in H_2O, Sigma Aldrich), FAU zeolite (Si/Al = 15, *Zeolyst* International, CBV-720), and Beta zeolite (Si/Al = 19, *Zeolyst* International, CP814C) were used as received.

S1.2 Catalyst Synthesis

Pd/FAU and Pd/beta were synthesized by incipient wetness impregnation (IWI) of Pd precursor onto zeolite support. Beta was pre-calcined in flowing air (breathing air, Airgas, 60 sccm g^{-1}_{sample}) at 773 K (2 K min^{-1} ramp rate, 2h). The precursor solution was prepared by dissolving the Pd precursor in deionized water (18.2 MΩ cm), where the concentration and volume of the Pd precursor solution were selected to achieve 0.5 wt.% Pd content while reaching the incipient point of the support. After impregnation, the catalyst was dried overnight in stagnant air at 333 K. The catalyst was then calcined in flowing air (breathing air, Airgas, 60 sccm g^{-1}_{sample}) at 773 K (2 K min^{-1} ramp rate) and held for 2 h. The calcined catalyst was then reduced in a quartz reactor by flowing H_2 (99.999%, Airgas, 50 sccm) at 523 K (2 $Kmin^{-1}$ ramp rate), followed by a 2 h hold, and then passivated in 1% O_2/Ar (UHP, Airgas) at ambient temperature for 0.5 h.

S1.3 Material Characterization

S1.3.1 Elemental Analysis

Metal loadings were determined by inductively coupled plasma optical emission spectroscopy (ICP-OES). Briefly, samples (15 mg) were placed in polyethylene bottles and dissolved in aqua regia, prepared by first adding 3.5 g HCl (ACS reagent, 37 wt.%, Sigma-Aldrich) followed by 2 g HNO_3 (Reagent Grade, 70 wt.%, Sigma-Aldrich). Then, 2.3 g HF (TraceMetalTM Grade, 50 wt.%, Fisher Chemical) was added, followed by the addition of 50 g de-ionized water after 24 h to dilute the acid to < 2 wt.% HF before elemental analysis. **Caution:** use appropriate personal protective equipment, ventilation, and engineering controls when working with HF. Calibrations were made using 1000 mg/L ICP standards (Fluka), after diluting to various extents using de-ionized water.

S1.3.2 Chemical Adsorption

N_2 adsorption was carried out on a Micromeritics 3-Flex instrument at 77 K using method reported previously by our group.[1] In a typical experiment, 50 mg of Pd/zeolite catalyst was first degassed under vacuum (~15 μmHg) at 673 K for 8 h on the Micromeritics VacPrep 061 sample degas system. Micropore volumes (sccm $gsample^{-1}$) were measured by analyzing the semi-log derivative plot of the adsorption isotherm ($\partial V_{ads} / \partial ln(P\ P_o^{-1})$) versus ($ln(P\ P_o^{-1})$). The maximum of ($\partial V_{ads} / \partial ln(P\ P_o^{-1})$) corresponds to the point at which the micropore filling occurs, and the minimum corresponds to the end of the micropore filling.[2] The volume of adsorbed gas (at STP) is converted into liquid micropore volume using a density conversion factor (0.001546) which uses the density of liquid N_2 at 77 K.

Static carbon monoxide (CO) chemisorption was carried out in a Micromeritics 3-Flex instrument at 308 K. In a typical CO chemisorption measurement, 100 mg of solid material was loaded into a quartz reactor, followed by degassing at 383 K (10 K min^{-1}) for 0.5 h and in situ reduction at 673

K in flowing H_2 for 0.5 h. The first adsorption isotherm was collected between 0 and 10 Torr (in increments of 0.5–1 Torr) to assess total CO uptake. The system was evacuated for 0.5 h to remove weakly adsorbed CO, and then a second isotherm was recorded to assess the reversible CO uptake. The irreversible CO uptake was determined by taking the difference between the total and reversible CO uptake, extracted by linearly extrapolating the difference between the isotherms to the limit of zero pressure. The metal dispersion (i.e., the fraction of exposed metal atoms) was then calculated by using Pd:CO stoichiometry of 1.5.[3] Errors on measured metal dispersions were estimated by setting the standard deviation of experimental values (from repeat measurements on 0.65 wt.% Pd/Al_2O_3 as a representative material) equal to the standard error.

S1.3.3 Transmission Electron Microscopy

High-resolution scanning transmission electron microscopy (STEM) was performed on FEI Titan G2 equipped with CEOS probe aberration corrector operated at 200 kV. A 200-mesh copper grid with mounted holey carbon films (Electron Microscopy Sciences) was placed face-down to a layer of powder with gentle pressure to pick up the particles. High-angle annular dark field (HAADF) images were collected and used for metal nanoparticle size measurement with a 23.4-mrad probe semi-angle and 140 pA probe current. A machine learning image processing tool (ParticleNN)[4] was used to automate the measurement of particle sizes. Particle size distributions were based on measurements of >200 particles for each sample, typically corresponding to 15-20 STEM images. The surface-area-weighted mean cluster diameter (d_{STEM}) estimated from STEM analysis by $d_{STEM} = \Sigma n_i d_i^3/\Sigma n_i d_i^2$.[5] The mean particle diameters were then reported as X ± Y nm, where X nm is the surface-weighted average particle diameter and Y nm is the standard deviation.

For fresh and spent Pd/Beta catalysts, among 15 total STEM images taken, 3 images showed a few larger Pd particles (5-10 nm), which were excluded from the particle size distributions shown in in Fig. 2. These particles presumably exist outside of zeolite nanopores. To assess whether these larger Pd particles could contribute to the overall Pd site density, the fraction of Pd surface area associated with these larger nanoparticles was estimated by comparing the projected area of the nanoparticles ($n_i d_{i\text{-large}}^2$) in the 3 STEM images containing such particles, to the total projected area of all Pd particles ($\Sigma n_i d_i^2$) in all 15 STEM images, assuming these images are representative of the overall statistical population of Pd particles on the material. This analysis showed that Pd surface sites on larger (> 5 nm) Pd particles account for <5% of the total Pd surface area over both fresh and spent Pd/Beta, suggesting that these smaller particles negligibly influence our catalytic reactivity results.

S1.3.4 Thermogravimetric analysis

Thermogravimetric analysis was carried out on TGA 550 or TGA 5500 instruments (TA instruments). 10-15 mg of solid material was placed into a platinum pan and loaded onto the instrument. In an O_2 atmosphere (50 ml min^{-1}), the sample was heated to 373 K at a ramp rate of 10 K min^{-1} with an isothermal hold of 0.33 h at 373 K to remove adsorbed water. Then, the temperature was ramped to 1073 K at 10 K min^{-1}.

S1.4 Reaction product analysis.

Reactants and products were quantified using Agilent Gas Chromatograph (GC; Agilent 8860) equipped with a Flame ionization detector (FID) with liquid injection via autosampler. A Restek

RTX-VMS capillary column (length: 30 m, ID: 250 μm, film thickness: 1.4 μm) was used. The injection port and FID were maintained at 513 K. The injection volume was 1 μL and a split ratio of 50 was used. Detailed information on feed and product calibrations, response factors, and confirmation of product identities using nuclear magnetic resonance (NMR) and GC-mass spectrometry (GC-MS) are provided in our previous work.[6]

S1.5 Reaction studies

Shape-selective probe reactions and N-LHC (de)hydrogenation batch reactions were carried out in 75 mL Parr Hastelloy high-pressure batch reactors (Parr Instrument Company). A PTFE (polytetrafluoroethylene) magnetic stir bar, an appropriate amount of catalyst, and feedstock were added to the reactor. The reactor was purged three times with 30 bar Ar followed by three purge cycles with 35 bar H_2. After purging, the reactor was pressurized to 35 bar H_2 for hydrogenation reactions or 7 bar Argon for dehydrogenation reactions. The heat-up time to the target temperature was 10 –15 minutes. The reaction mixture was stirred at 750 rpm. For shape-selective probe reactions (using toluene or TIPB), 4-5 samples were collected using a dip-tube to measure the reaction time course. At the end of the reaction, the reactor was quenched in ambient temperature water for 0.5 h, depressurized, and opened. The reaction products were filtered using a 0.22 μm PTFE syringe filter before analysis. Post-reaction, catalysts were filtered and washed 3× with n-hexane (1 ml per 100 mg catalyst), then analyzed by TGA to quantify coke formed during reaction. Catalysts were calcined (in air) and reduced (in H_2) at 673 K, followed by CO chemisorption to assess changes to active metal surface area; selected spent catalysts after calcination at 673 K and reduction at 523 K were further analyzed by STEM imaging to assess metal particle size distributions.

Adsorption of N-MID and 8H-NMID on the used supports was quantified by mixing 40 mg of the support material with 1 wt.% N-LHC in dodecane solvent (3 ml) in a glass vessel under ambient conditions and stirring on a magnetic stir plate. A liquid aliquot was collected after mixing for 24 h, filtered using a 0.22 μm PTFE filter, and analyzed using GC to quantify the fractional uptake of N-LHC on a given support (**Figure S8**). Equation 6b was used to correct the carbon balances for the batch reactor under dilute conditions. After these adsorption tests, the support was filtered and washed with n-hexane, which removed reversibly adsorbed indoles. Irreversibly adsorbed indoles on the supports were assessed by post-adsorption TGA (**Figure S11,** and **Table S4**), the quantity of which was subtracted from post-reaction TGA measurements to quantify coking induced by N-LHC hydrogenation reactions.

Given that adsorption measurements were done at ambient temperature while batch reactions are conducted at 423-453 K, this calculation assumes that the extent of support adsorption is independent of temperature. As shown in Fig. S8, this approach results in (adjusted) carbon balances of >97% for dilute NMID hydrogenation reactions, suggesting this is a reasonable approach to account for the effect of support adsorption. We note that adsorption of NMID does not significantly affect flow reactor data (as the support surface adsorbs indoles during the transient startup period) or batch reaction data at higher NMID concentrations (as the support adsorption becomes a negligible fraction of total NMID). For these reasons, although the extent of NMID adsorption may change with temperature, such effects would not change the conclusions of this article and therefore did not explore this effect further.

Continuous flow reactions were performed in a laboratory fixed-bed continuous flow reactor; details are given in our previous work.[6] Appropriate catalyst masses (3-25 mg), sieved to retain aggregates of size between 180-250 μm, were packed in between two pieces of glass wool. Temperature control of the reactor was achieved by using aluminum heating blocks with a ¼" cylindrical annulus to surround the reactor; resistive heating tape (Briskheat) was wrapped around the heating block and connected to a temperature controller (Love Controls). A thermocouple (Omega) was placed at the center of the reactor via a cut-out slot in the aluminum heating block. The system was then insulated using fiberglass insulation. Inlet gas flows were maintained using mass flow controllers (Brooks). The system pressure was controlled using a back-pressure regulator (Swagelok). The liquid feed was delivered via a High-performance liquid chromatography (HPLC) pump (Eldex). The liquid and gas mixture then flowed upward through the reactor, and the liquid-phase products were collected downstream in a 120 mL stainless steel condenser (Swagelok) immersed in water at ambient temperature (permanent gases passed through this condenser and through the back-pressure regulator to be vented). Product samples were collected at 1-2 h intervals and were filtered using a 0.2 μm PTFE syringe filter before analysis.

Prior to flow reactions, catalysts were reduced *in situ* under flowing H_2 at 523 K to ensure a zero-valent metallic state before rate and stability measurements. N-MID hydrogenation reactions were carried out using a 1 wt.% solution of N-LHC in dodecane (1.8 ml h^{-1}), where dodecane was chosen due to its inertness under reaction conditions and relatively high boiling point. After flow reactions, spent catalysts were washed with flowing dodecane (15 ml), and n-hexane (70 ml) and then dried overnight under flowing Ar. For regeneration following continuous flow reactions, catalysts were calcined (673 K, 2 K min^{-1}, 50 sccm air, 1 h hold) and re-reduced (523 K, 2 K min^{-1}, 50 sccm H_2, 1 h hold) *in situ*.

Conversion, selectivity, and yields were calculated using **Equations S1-3**. The reaction rate was defined as the moles of product per surface metal site (quantified by CO chemisorption) per unit time (**Equation S4**). We note that rate represent an average value across all exposed metal surface sites (N_s) counted by CO chemisorption (particularly if the reaction is structure-sensitive, which we did not investigate in detail here).[7] The cumulative catalytic turnover number, defined as the moles of product per mole surface active site (on the fresh catalyst) in the catalytic reaction, was calculated using **Equation S5.** We calculated turnover numbers in both continuous flow reactions (**Equation S5a**, by integrating the rate across TOS) and batch reactions (**Equation S5b**). The carbon balance of reaction was estimated using **Equation 6a**, and later corrected for support adsorption using **Equation S6b**.

$$X_{N-LHC} = (C^{in}_{N-LHC} - C^{out}_{N-LHC})/C^{in}_{N-LHC} \times 100 \quad \textbf{(Eqn. S1)}$$

$$Y_{2H-Indole} = C_{2H-Indole}/C^{in}_{N-LHC} \times 100 \quad \textbf{(Eqn. S2)}$$

$$S_{2H-Indole} = C_{2H-Indole}/(C^{in}_{N-LHC} - C^{out}_{N-LHC}) \times 100 \quad \textbf{(Eqn. S3)}$$

$$rate = [(Y_{2H-Indole})+(Y_{4H-Indole})+(Y_{8H-Indole})] \times F^{in}_{indole}/N_s \quad \textbf{(Eqn. S4)}$$

$$TON_{Flow\ Reactor} = \int_0^{TOS} rate\ d(TOS) \quad \textbf{(Eqn. S5a)}$$

$$TON_{Batch\ Reactor} = (Y_{2H-Indole}+Y_{4H-Indole}+Y_{8H-Indole}) \times M_{N-LHC}/N_s \quad \textbf{(Eqn. S5b)}$$

$$Carbon\ balance = \frac{C_{N-LHC}^{product}}{C_{N-LHC}^{in}} \quad \textbf{(Eqn. S6a)}$$

$$Adjusted\ Carbon\ balance = \frac{C_{N-LHC}^{product} + C_{N-LHC}^{adsorbed}}{C_{N-LHC}^{in}} \quad \textbf{(Eqn. S6b)}$$

Where, $C_{N\text{-}LHC}$ is the molar concentration of N-LHC, F_{indole}^{in} is the inlet molar flow rate of indole, N_s is the moles of surface metal sites added in the reactor, 'rate' is the reaction rate at a given time-on-stream (TOS), $TON_{Flow\ Reactor}$ is the cumulative turnover number in a flow reaction following a given amount of time-on-stream (TOS), $TON_{Batch\ Reactor}$ is the total turnovers performed by the catalyst in a batch reaction following a given reaction time, and $M_{N\text{-}LHC}$ is the moles of indole feed introduced in a batch reactor.

A first-order deactivation model (**Equation S7**) was fitted to the reaction data and assessed initial reaction rates (fits to this model are shown in the SI, **Figure S6**). Experimental errors were estimated as 95% confidence intervals from the uncertainty in the Y-intercept of the first-order deactivation model fits. The estimated 1st order deactivation rate constants are then given in the form of X ± Y (Table 2), where X is the mean value of the deactivation rate constant and Y is the standard error. The site-loss turnover number (SLT) was defined as the moles of product per mole of surface sites lost, as calculated using **Equation S8**. Mathematically, this metric is the inverse of the cumulative site-loss selectivity discussed by Bhan et al.[8] and provides an estimate of the total turnovers a surface metal site could undergo before complete deactivation, assuming that deactivation occurs non-selectively (i.e., all sites are equivalent in their deactivation behavior).

$$ln\,(rate) = ln\,(rate_{,t=0}) - k_d t \quad \textbf{(Eqn. S7)}$$

$$Site - loss\ turnover\ number\ (SLT) = (\Delta TON/\Delta t)/(\Delta n_*/\Delta t) \quad \textbf{(Eqn. S8)}$$

Where k_d is the first-order deactivation rate constant and n_* is the instantaneous fraction of active sites remaining (**Equation S9**), estimated from the fractional decrease in reaction rate as a function of TOS.

$$n_* = 1 - \frac{rate_{min,TOS}}{rate_{max}} \quad \textbf{(Eqn. S9)}$$

$rate_{min,t}$ gives the minimum reaction rate at a given TOS (taken as the rate at the final TOS in a given continuous flow experiment), and $rate_{max}$ is the maximum measured reaction rate in the same experiment, which occurred at or near the beginning of the experiment (at early TOS). Experimental error in SLT values (reported in Table 2) was propagated by using the known errors on rate measurements in Eqn. S9 to assess the error in the remaining fraction of active sites, n_*.

S2. Characterization of catalyst materials and reaction products

S2.1. Catalyst characterization.

Table S1. *Physical and Chemical Properties of supported catalysts*

Catalyst	Micropore Volume / (cm^3/g)	Metal Loading / (wt. %)	Metal Dispersion [a] / (%)	d_{STEM} [b] / (nm)
**Pd/SiO_2**	-	0.54	35	2.1 ± 0.7
**Pd/Al_2O_3**	-	0.65	55	0.98 ± 0.5
**Pd/FAU**	0.28	0.51	28	1.50 ± 0.8
**Pd/Beta**	0.23	0.52	25	1.27 ± 0.5

[a] Metal dispersion estimated by CO chemisorption
[b] Surface- area-averaged mean cluster diameter.

N_2 physisorption measurements of Pd/zeolites indicated micropore volumes of 0.28 cm^3/g (Pd/FAU)[9] and 0.23 cm^3/g (Pd/Beta)[10], respectively, consistent with literature-reported values for parent FAU and Beta zeolites. Comparing adsorption and desorption branches, the lack of hysteresis implies the absence of significant mesoporosity in these materials.

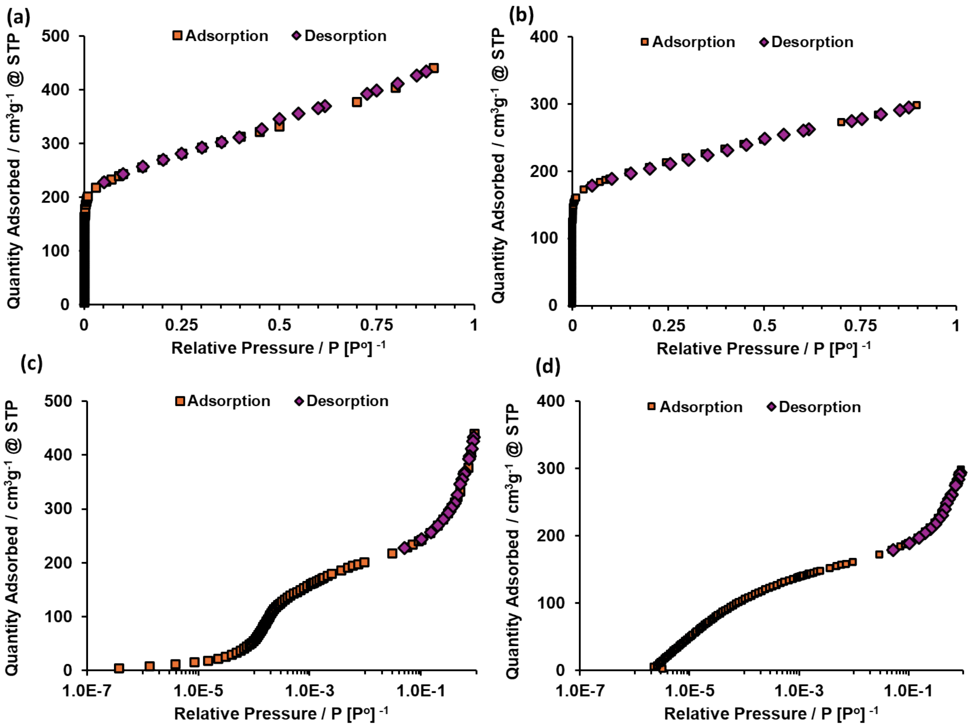

Figure S1. *N_2 adsorption isotherms on (a,c) Pd/FAU, (b,d) Pd/Beta.*

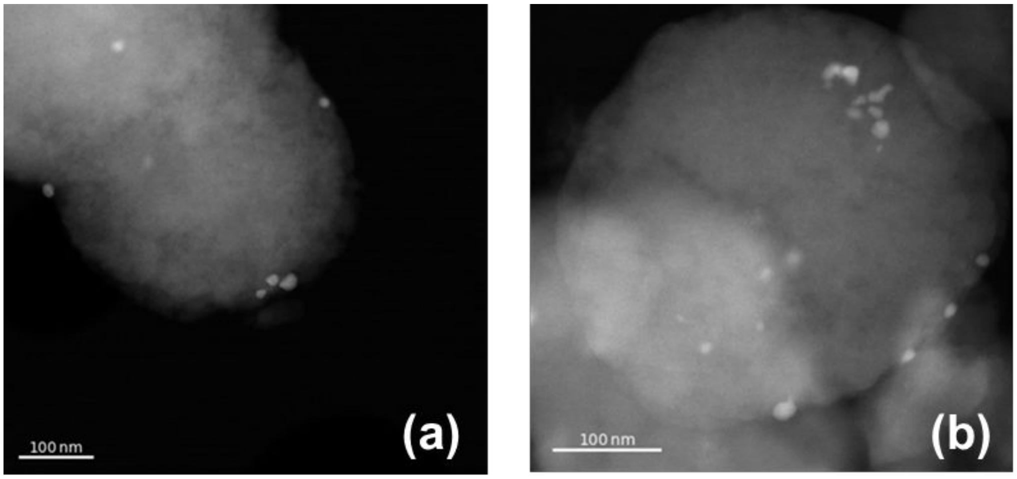

Figure S2. *Representative STEM image showing larger Pd particles (5-10 nm) observed in a few of the STEM images taken of (a) Fresh Pd/Beta and (b) Spent, Regenerated Pd/Beta (reaction data is given in Figure S8). These particles accounted for <5% of the total Pd surface area over both fresh and spent Pd/Beta.*

S2.2. Shape-selective probe reactions to assess extent of Pd encapsulation in zeolites.

We performed shape-selective probe reactions with toluene and 1,3,5-trisopropylbenzene (TIPB) to determine the extent of Pd encapsulation in zeolite pores, following reported approaches (Table 1).[11,12] Toluene (kinetic diameter: 5.9 Å)[13] can access both external and zeolite-confined Pd sites, whereas TIPB (kinetic diameter: 9.5 Å)[14] likely cannot diffuse into the nanopores of FAU and Beta zeolites. As a result, rates of toluene hydrogenation should reflect the reactivity of all Pd sites in the material, while TIPB hydrogenation should reflect only the reactivity of Pd sites located on the external surface of the zeolite (and/or in larger mesoporous voids). Rates of toluene/TIPB hydrogenation were measured from batch reactor time course experiments at <30% reactant conversion, with linear fits shown in **Figure S3**. Reaction rates were defined as moles of reactant converted per time normalized by the exposed metal surface sites quantified by CO chemisorption. Hydrogenation rates of toluene (and in some cases, TIPB) were higher on Pd/zeolites compared to Pd/SiO_2, which may reflect influences such as promotional effects of zeolitic acid sites[15–17] and/or pore confinement[18] on hydrogenation rates. The ratio of reaction rates for toluene versus TIPB (χ) on Pd/SiO_2 establishes a baseline for comparison that reflects differences in reactivity between these molecules in the absence of shape-selectivity effects. This ratio, χ (Eq 1), was then measured over Pd/zeolite catalysts. The ratio of χ values of Pd/zeolite versus Pd/SiO_2 is defined as φ (Eq 2). The fraction of encapsulated Pd sites is then calculated from φ in **Eqn. S10**, where the "1" in this estimation comes from the unconfined control catalyst. In shape-selective probe reactions, we assume that hydrogenation rates are structure-insensitive[19,20] and that any more complex effects of support identity on hydrogenation rates affect toluene and TIPB conversion to similar extents.

$$\frac{\varphi_{Pd/Zeolite} - 1}{\varphi_{Pd/Zeolite}} = fraction\ Pd\ Encapsulated \qquad (\boldsymbol{Eqn.\ S10})$$

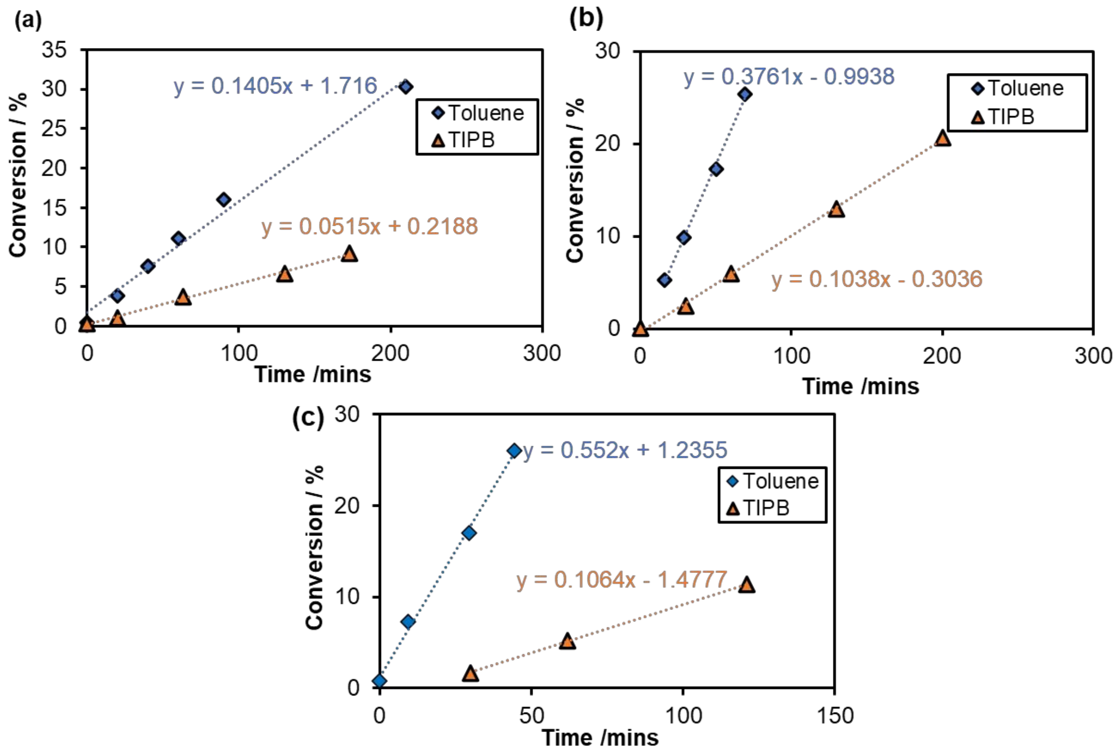

Figure S3. *Shape-selective hydrogenation probe reactions. (a) Pd/SiO_2, (b) Pd/FAU, (c) Pd/Beta. Reaction Conditions: 82mM of toluene or 1,3,5 tri-isopropylbenzene (TIPB) in dodecane (55mL), 423 K, 35 bar H_2. Catalyst masses: (a) Toluene – 80 mg, TIPB – 150 mg, (b) Toluene – 45 mg, TIPB – 350 mg, and (c) Toluene – 14 mg, TIPB – 72 mg.*

S3. Continuous flow reactions.

S3.1 Product distribution in continuous flow reactions.

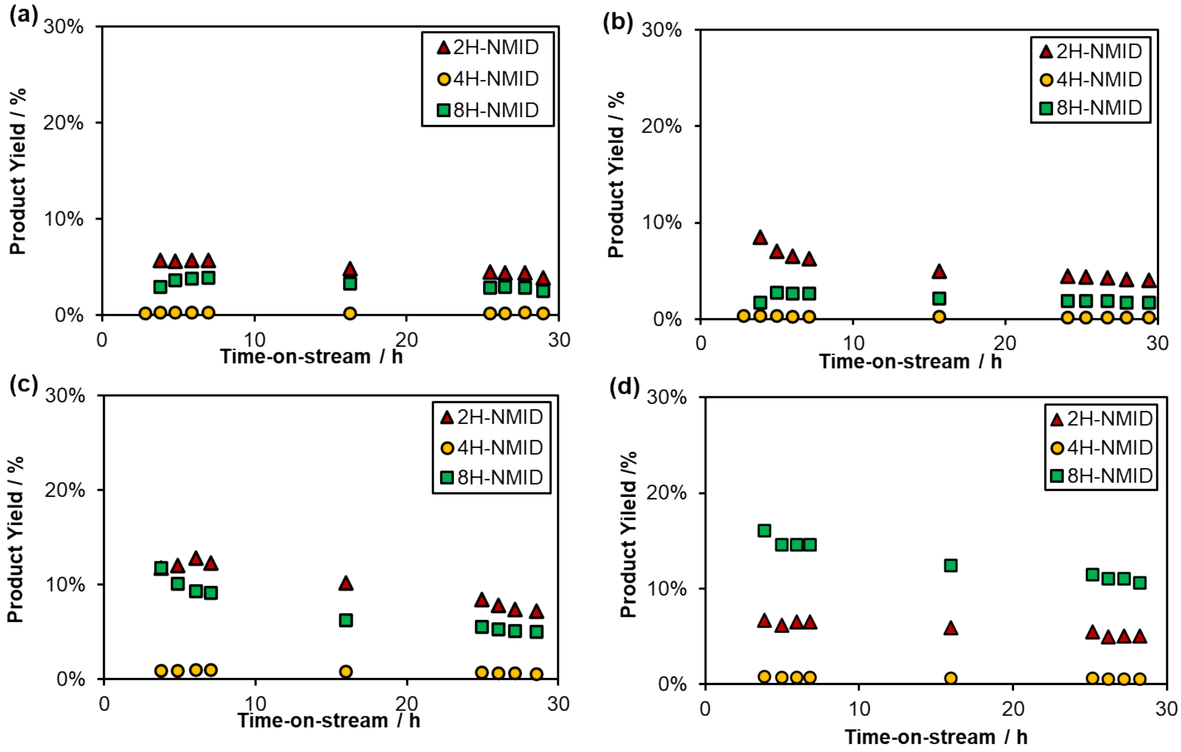

Figure S4. *Product yields during hydrogenation of N-MID: (a) Fresh Pd/Beta, (b) Regenerated Pd/Beta, (c) Fresh Pd/FAU, (d) Regenerated Pd/FAU. Rates versus time-on-stream data are shown in Figure 1. Product yields over Pd/SiO_2 and Pd/Al_2O_3 are reported in our previous work.*[6] *The N-MID hydrogenation reaction network is shown in Scheme 1.*

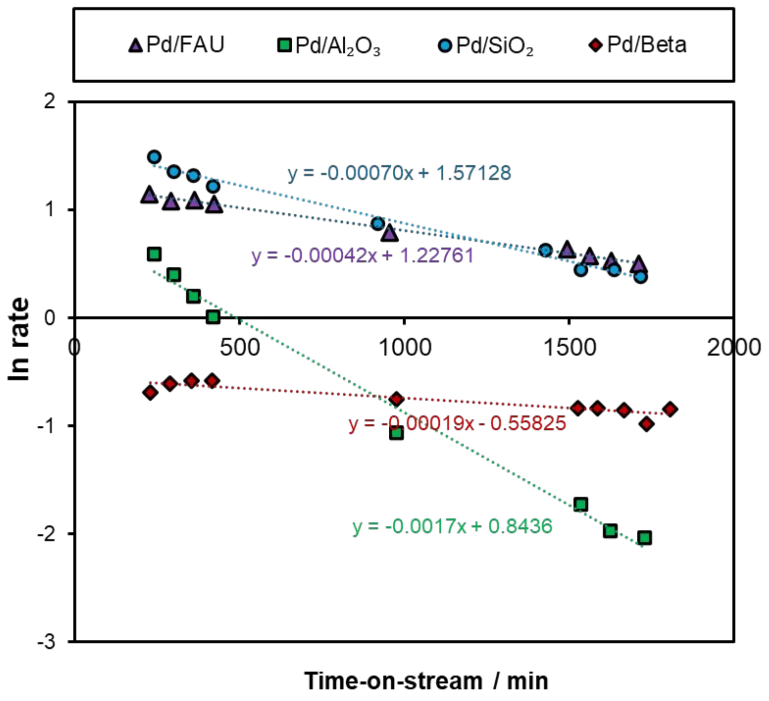

Figure S5. *First-order deactivation fits for N-MID hydrogenation using equation S7, and reaction data on fresh catalysts given in Figure 1. Rate is reported in units of [mol_{prod} $(mol_{Pd,surface})^{-1}$ $(min)^{-1}$].*

S3.2 Assessing the influence of transport limitations on measured rates.

External mass transport limitations. To assess the influence of external mass transport limitations on measured rates of 1 wt.% N-MID hydrogenation, we evaluated Mears' Criterion for the 0.54 wt.% Pd/SiO_2 catalyst in our previous work, considering the expected rate of mass transfer of N-MID to the catalyst surface relative to the measured rate of reaction.[6] Our analysis showed that external N-MID mass transport limitations are negligible under the studied conditions; because Pd/SiO_2 had the highest rate among catalyst studied here, this analysis also excludes N-MID transport limitations for the Pd/zeolites studied in this work.

To further exclude the possibility of interphase transport limitations including those associated with gas-to-liquid transport of H_2 and with liquid-to-catalyst transport of liquid-phase species (dissolved H_2 and N-MID), we performed a continuous flow hydrogenation reaction of 1 wt.% N-MID over the 0.52 wt.% Pd/Beta catalyst (similar to that in Figure 1d) but with varying liquid or gas flow rates (**Figure S6, Table S2**). Reaction rates depend weakly on H_2 gas flow rate (varied by 3× from to 10 to 30 sccm) or the liquid flow rate of 1 wt.% N-MID (varied by 3× from 1.8 to 5.4 ml hr^{-1}), confirming that rates are free of significant interphase transport limitations within the experimental uncertainty of our rate measurements.

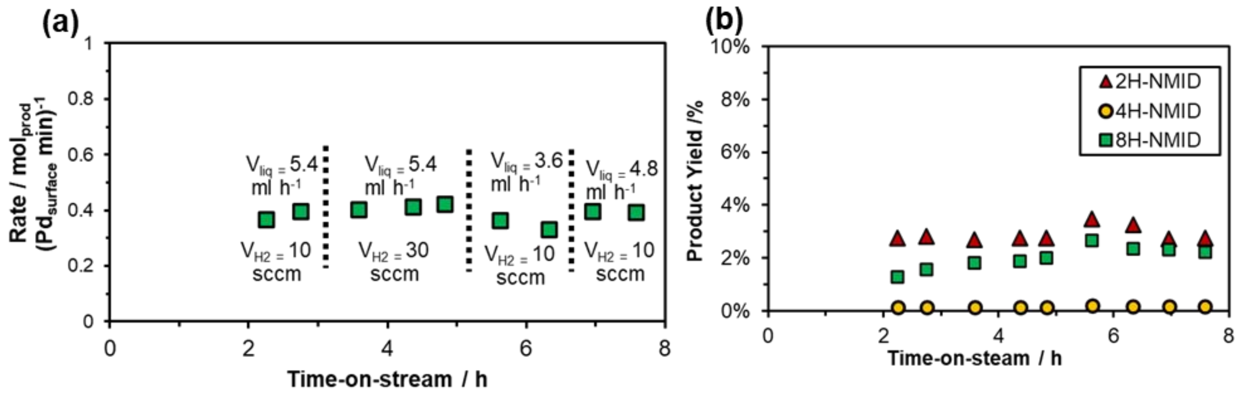

Figure S6. *Hydrogenation of 1 wt.% N-MID in dodecane in a continuous flow reactor over 0.52 % Pd/Beta: (a) liquid feed flow rate or H_2 gas flow rate varied periodically with time-on-stream, as indicated in the figure [373 K, 50 mg of 0.5 wt.% Pd/Beta, 35 bar H_2]; (b) Product yields versus time-on-stream during hydrogenation of 1 wt.% N-MID in dodecane shown in Figure S6a.*

Table S2. *Rates of 1 wt.% NMID hydrogenation (extracted from Figure S6) at various liquid and gas flow rates, with associated product distributions. *initial rate measured in a separate experiment with 25 mg of 0.5 wt% Pd/Beta, from Figure 1d.*

Liquid flow rate/ ml h^{-1}	H_2 flow rate / sccm	Rate / mol_{prod} $(Pd_{surface}$ $min)^{-1}$
5.4	10	0.38
5.4	30	0.41
3.6	10	0.36
4.8	10	0.40
1.8*	10*	0.59*

Internal mass transport limitations. The Weisz-Prater criterion was used to estimate the impact of intracrystalline mass transport limitations on rates of 1 wt.% N-MID hydrogenation over Pd/Beta, as described by Equation S11.[21]

$$N_{WP} = \frac{r'_{obs} * \rho_c * r_p^2}{D_{eff} * C_B} \qquad \textbf{(Eqn. S11)}$$

Where:

$r'_{obs} = 1.2 \times 10^{-7}$ mol g^{-1} sec^{-1} per-mass reaction rate for Pd/Beta during N-MID hydrogenation at 35 bar H_2 and 373 K (Figure 1d).

$\rho_c = 1.60$ g cm^{-3}.[22]

r_p = 0.25 μm measured by Bhan and co-workers for the commercial parent Beta zeolite (Zeolyst: CP-814C) using SEM.[23]

C_B = Bulk N-MID concentration = 57.3 mmol L^{-1}, assuming no external transport limitations.

D_{eff} = effective diffusivity.

Because the effective diffusivity of N-MID in zeolite pores has not been reported to our knowledge, we used the reported effective diffusivities of two aromatic molecules similar in size to indoles: toluene (kinetic diameter = 5.9Å)[13] and o-xylene (kinetic diameter = 6.8Å).[24] As an order-of-magnitude estimate, we assumed that the effective diffusivity of N-MID in the catalyst pores is similar to the effective diffusivity of toluene ($3.0*10^{-10}$ cm^2 s^{-1})[25] or o-xylene ($2.9*10^{-10}$ cm^2 s^{-1}) at 373 K, with D_{eff} values reported by Corma and co-workers in H-Beta (Si/Al = 18).[25] Based on these estimated values, **N_{WP} = 0.003**, suggesting the absence of significant intracrystalline mass transport limitations in Pd/Beta. Given the larger pore size of FAU relative to Beta, we expect that transport limitations are likely also negligible on Pd/FAU. Given the low Pd loadings studied here (0.5 wt.%), we expect that the presence of Pd nanoparticles likely does not significantly hinder transport.

S4. Batch reactor (de)hydrogenation of N-LHCs and post-reaction catalyst characterization.

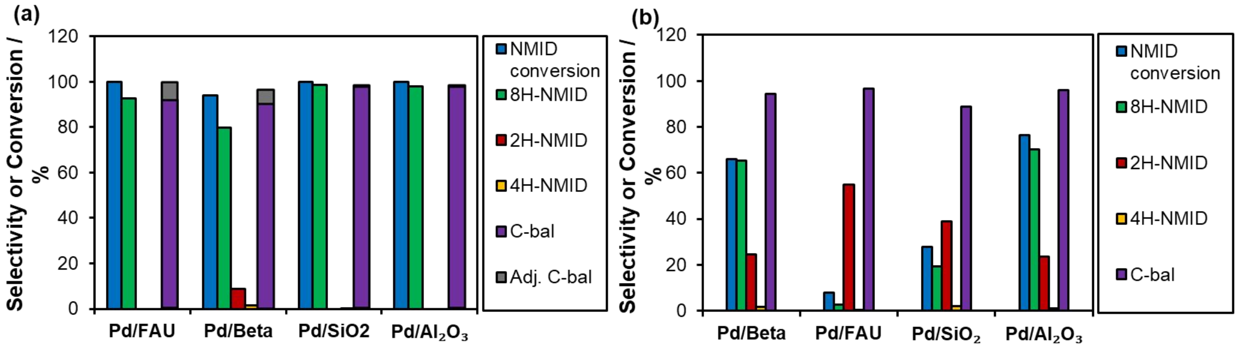

Figure S7. *Hydrogenation of (a) 2 wt.% N-MID in dodecane or (b) pure N-MID in a batch reactor over various supported Pd catalysts. Reaction conditions: (a) 45ml, 750 rpm, 35 bar H_2, at 423 K for 22 h (for Pd/SiO_2, reaction time was 10 h). Catalyst masses: 200 mg of 0.5% Pd/FAU, 300 mg of 0.5% Pd/SiO_2, and 0.5% Pd/Beta. (b) 15 ml, 35 bar H_2 at 423 K for 90 h. Catalyst masses: 300 mg of 0.5% Pd/FAU, 0.5% Pd/SiO_2, 0.5% Pd/Beta, and 0.7% Pd/Al_2O_3. "Adj. C- bal" is the adjusted carbon balance after accounting for carbon lost via support adsorption (Figure S8), as given in equation S6b. The reaction network is shown in Scheme 1.*

Table S3. *N-MID conversion and cumulative catalytic turnover numbers during hydrogenation pure N-MID over supported Pd catalysts shown in Figure S7b.*

Catalyst	Conversion / %	Catalytic Turnover Number / mol_{prod} $mol_{Pd,surface}^{-1}$
**Pd/Beta**	66.1	62,800
**Pd/FAU**	7.9	2,100
**Pd/SiO_2**	27.9	7,700
**Pd/Al_2O_3**	76.4	8,700

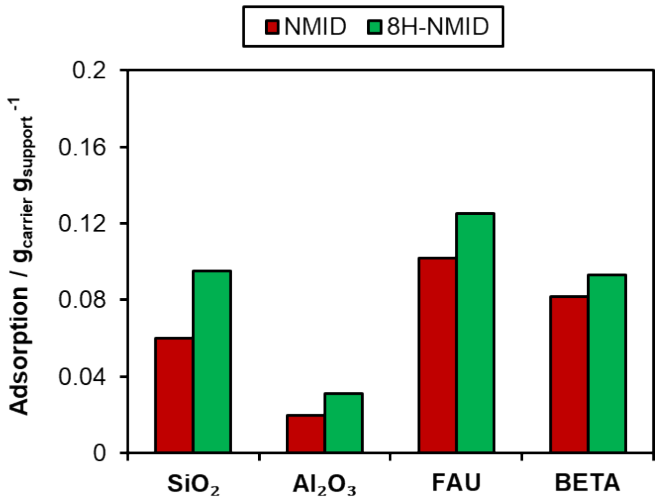

Figure S8. *Mass of N-MID or 8H-NMID adsorbed onto a given catalyst support, relative to support mass, measured in control experiments by mixing 40 mg of support with 1 wt. % N-MID or 8H-NMID in dodecane (3 ml) at ambient temperature for 24 h.*

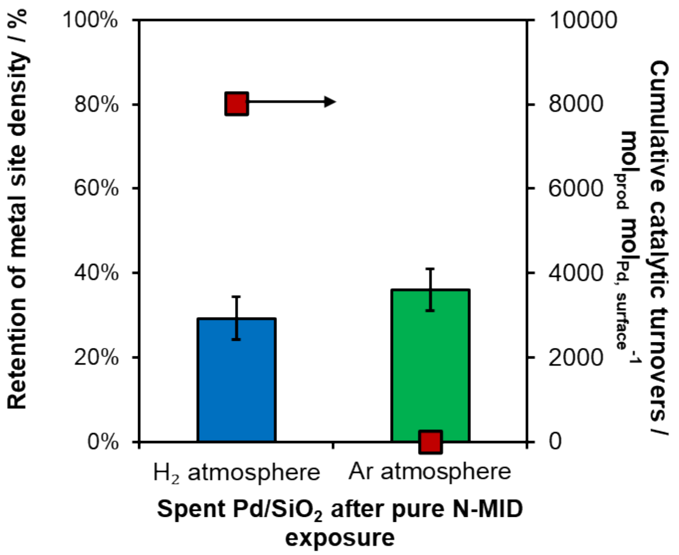

Figure S9. *Percent retention of metal site density over Pd/SiO_2 (by CO chemisorption) following exposure to pure N-MID in presence and absence of H_2 (Control experiment). Conditions: 300 mg of 0.5 wt. % Pd/SiO_2, 15 ml pure N-MID, 35 bar H_2 or 25 bar Ar, 423 K, 90 h.*

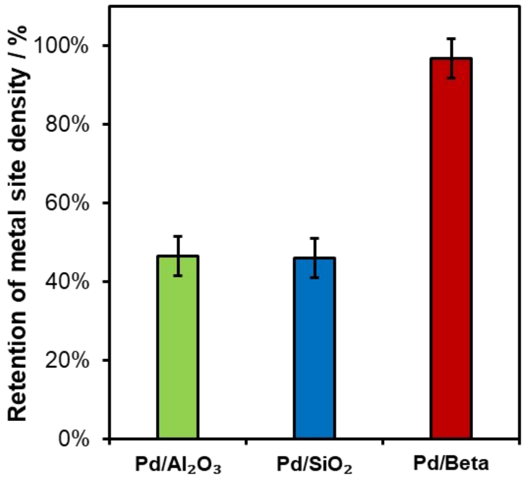

Figure S10. *Percent retention of metal site density over Pd/SiO_2, Pd/Al_2O_3, and Pd/Beta (by CO chemisorption) following exposure to 8H-NMID in presence of H_2 (Control experiment). Conditions: 300 mg of 0.5 wt. % Pd/SiO_2, 0.8 wt.% Pd/Al_2O_3, 0.5 wt.% Pd/Beta, 15 ml pure 8H NMID, 35 bar H_2, 453 K, 90 h.*

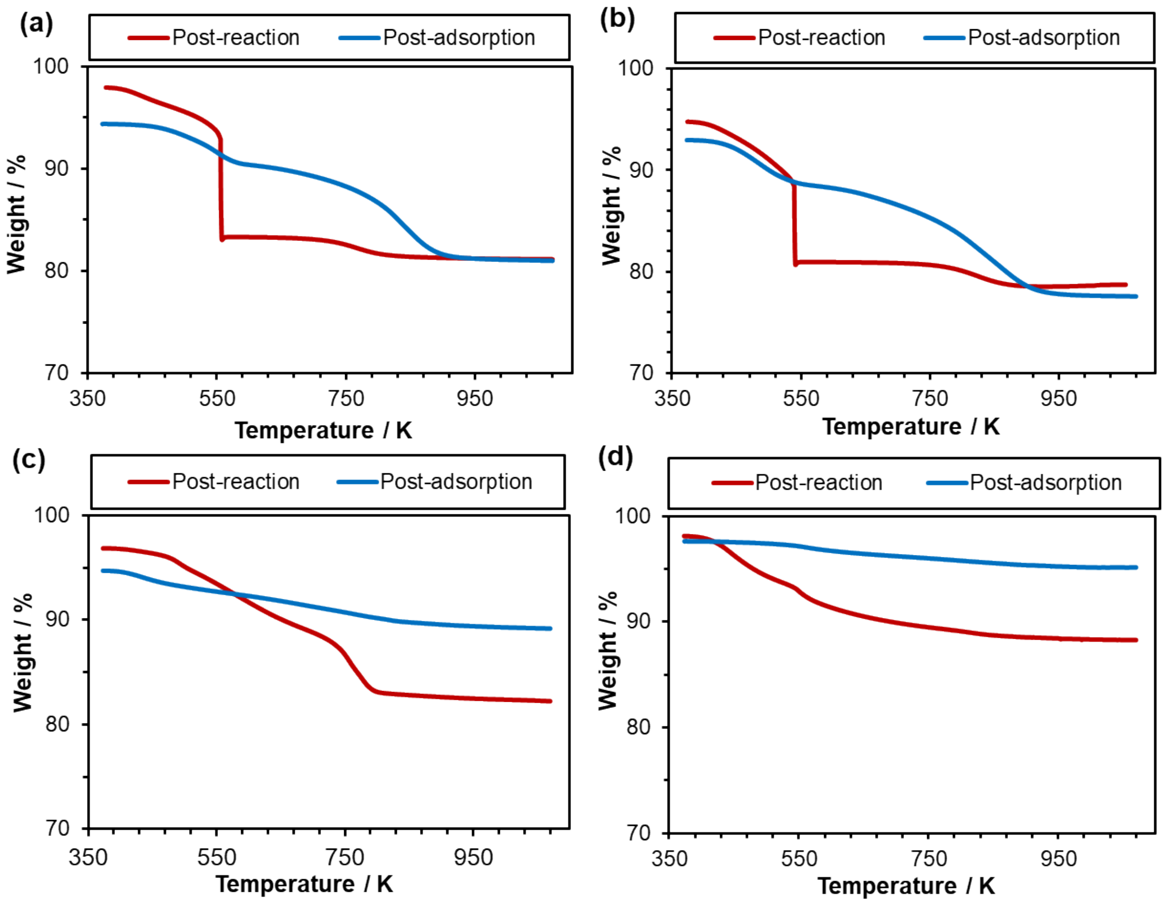

Figure S11. *Thermogravimetric analyses over supported Pd catalysts after N-MID (2 wt.%) hydrogenation: (a) Pd/Beta, (b) Pd/FAU, (c) Pd/Al_2O_3, (d), Pd/SiO_2. Post-reaction catalysts were generated from the reaction results given in Figure S7a. Details of adsorption experiments are mentioned in section S1.4. For comparison, Pd/FAU, Pd/Beta, Pd/SiO_2, and Pd/Al_2O_3 performed 2100, 2800, 1230, and 780 catalytic turnovers, respectively.*

Table S4. *Quantifying coke deposition on supported Pd catalysts during N-MID hydrogenation (or adsorption) using thermogravimetric analysis. Weight loss was estimated from the change in weight in the temperature range 373–1073 K, excluding the weight lost at <373 K attributed to water. Data is given in Figure S11. The extent of coking is estimated by subtracting the weight loss of the post-adsorption samples from that of the post-reaction catalysts, thereby excluding the contribution of support adsorption from the quantification of coke formation during reaction.*

Catalyst/ Support	Weight lost from bare support in control experiment (wt. %)	Weight lost post-reaction (wt. %)	Extent of coking (wt. %)
Beta	14.1		
Pd/Beta		17.2	3.1
FAU	16.6		
Pd/FAU		19.6	3.0
Al_2O_3	5.84		
Pd/Al_2O_3		15.10	9.3
SiO_2	2.53		
Pd/SiO_2		10.03	7.5

Table S5. *Metal loading of fresh and spent Pd catalysts determined via ICP-OES*

Catalyst	Fresh	Spent		
		N-MID (post reaction)[1]	N-MID (in Ar, no reaction)[2]	8H-NMID (in H_2, no reaction)[3]
	Metal Loading / wt. %	Metal Loading / wt. %	Metal Loading / wt. %	Metal Loading / wt. %
Pd/SiO_2	0.53	0.40 (25% leaching)	0.42 (21% leaching)	0.51 (4% leaching)
Pd/FAU	0.51	0.54	-	-
Pd/Beta	0.52	0.58	-	-

Note: [1]Experimental conditions given in Figure S7b. [2]Spent catalyst following control experiment using N-MID in presence of Ar; detailed conditions given in Figure S9. [3]Spent catalyst following control experiment using 8H-NMID in presence of H_2; detailed conditions given in Figure S10.

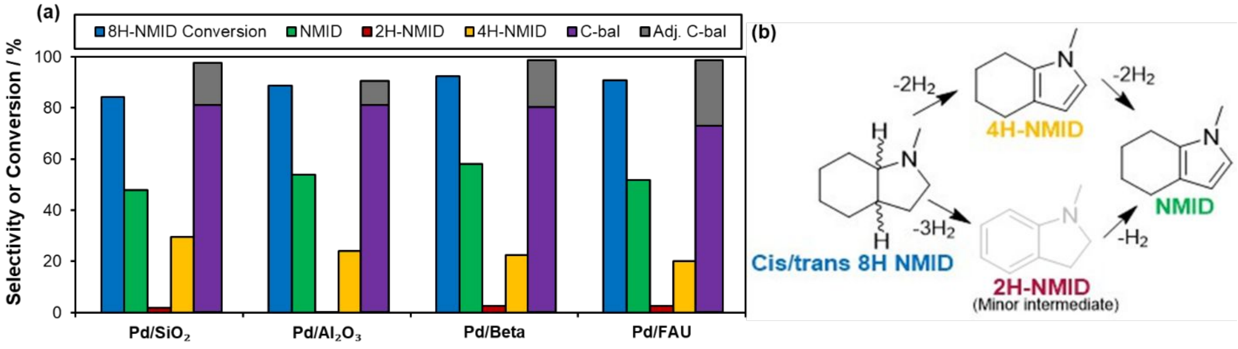

Figure S12. (a) *Dehydrogenation of 1 wt.% 8H N-MID in dodecane (with a feedstock cis/trans ratio of 2.0) in a batch reactor over supported Pd catalysts. Reaction conditions: 15ml, 7 bar Argon, 453 K, 3 h, 750 rpm. Catalyst masses: 200 mg of 0.5% Pd/FAU, 0.5% Pd/SiO_2, 0.7% Pd/Al_2O_3, and 0.5% Pd/Beta. "Adj. C- bal" is the adjusted carbon balance after accounting for carbon lost via support adsorption (Figure S8), as given in equation S6b. (b) Reaction network for dehydrogenation of 8H-NMID over supported Pd catalysts.*

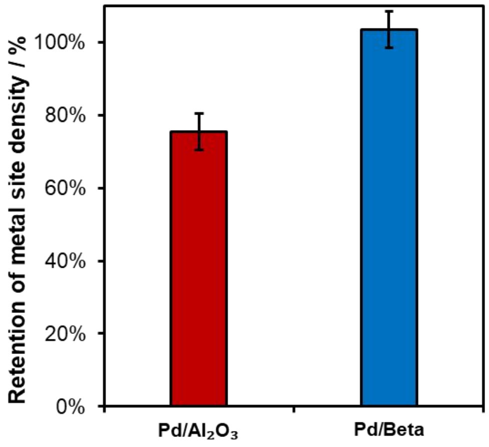

Figure S13. *Percent retention of Pd site density of Pd/Al_2O_3 and Pd/Beta (by CO chemisorption) following dehydrogenation of 1 wt.% 8H-NMID in dodecane in a batch reactor, followed by regeneration via calcination at 673 K in air. Reaction conditions and product distributions are given in Figure S12.*

Table S6. *Metal dispersion of spent, regenerated catalysts (measured via CO chemisorption) following exposure to N-LHCs (N-MID or 8H-NMID) under different gas environments (H_2 or Ar). Results in this table provide metal dispersions of spent catalysts corresponding to various experiments throughout the paper where the percent retention of Pd site density was reported.*

Catalyst	N-LHC	Concentration	Gas Environment	Metal Dispersion / %
**Pd/SiO₂**	N-MID	2 wt.%	H_2	21.2
**Pd/Al₂O₃**	N-MID	2 wt.%	H_2	19
**Pd/FAU**	N-MID	2 wt.%	H_2	28.2
**Pd/Beta**	N-MID	2 wt.%	H_2	24
**Pd/SiO₂**	N-MID	Pure	H_2	10.3
**Pd/Al₂O₃**	N-MID	Pure	H_2	6.7
**Pd/FAU**	N-MID	Pure	H_2	18.2
**Pd/Beta**	N-MID	Pure	H_2	22.1
**Pd/SiO₂**	N-MID	Pure	Ar	12.1
**Pd/SiO₂**	8H-NMID	Pure	H_2	16.1
**Pd/Beta**	8H-NMID	Pure	H_2	24.2
**Pd/Al₂O₃**	8H-NMID	Pure	H_2	24.8
**Pd/Al₂O₃**	8H-NMID	1 wt.%	Ar	41.8
**Pd/Beta**	8H-NMID	1 wt.%	Ar	26.0

References for Supplemental Information.

(1) Brungardt, E. A.; Sunkireddy, V.; Perez-Aguilar, J. E.; Krishna, S. H. Elucidating Redox Pathways for N2O Selective Catalytic Reduction with NO and NH3 over Fe-Chabazite Zeolites. *Applied Catalysis B: Environment and Energy* **2025**, *362*, 124708. https://doi.org/10.1016/j.apcatb.2024.124708.

(2) Borghard, W. S.; Reischman, P. T.; Sheppard, E. W. Argon Sorption in ZSM-5. *Journal of Catalysis* **1993**, *139* (1), 19–23. https://doi.org/10.1006/jcat.1993.1002.

(3) Krishna, S. H.; McClelland, D. J.; Rashke, Q. A.; Dumesic, J. A.; Huber, G. W. Hydrogenation of Levoglucosenone to Renewable Chemicals. *Green Chem.* **2017**, *19* (5), 1278–1285. https://doi.org/10.1039/C6GC03028A.

(4) Matveev, A. V.; Nartova, A. V.; Sankova, N. N.; Okunev, A. G. DLgram Cloud Service for Deep-learning Analysis of Microscopy Images. *Microscopy Res & Technique* **2024**, *87* (5), 991–998. https://doi.org/10.1002/jemt.24480.

(5) Bergeret, G.; Gallezot, P. Particle Size and Dispersion Measurements. In *Handbook of Heterogeneous Catalysis*; Ertl, G., Knözinger, H., Schüth, F., Weitkamp, J., Eds.; Wiley, 2008; pp 738–765. https://doi.org/10.1002/9783527610044.hetcat0038.

(6) Ahsan, S.; Edgar, M. D.; Chanthachaiwat, S.; Wei, J.; Voyles, P. M.; Krishna, S. H. Reactant-Dependent Stability of Supported Metal Catalysts for Hydrogen Storage in N-Heterocyclic Carriers. *Chemical Engineering Journal* **2025**, *519*, 164467. https://doi.org/10.1016/j.cej.2025.164467.

(7) Boudart, M. Turnover Rates in Heterogeneous Catalysis. *Chem. Rev.* **1995**, *95* (3), 661–666. https://doi.org/10.1021/cr00035a009.

(8) Foley, B. L.; Johnson, B. A.; Bhan, A. A Method for Assessing Catalyst Deactivation: A Case Study on Methanol-to-Hydrocarbons Conversion. *ACS Catal.* **2019**, *9* (8), 7065–7072. https://doi.org/10.1021/acscatal.9b01106.

(9) Galarneau, A.; Mehlhorn, D.; Guenneau, F.; Coasne, B.; Villemot, F.; Minoux, D.; Aquino, C.; Dath, J.-P. Specific Surface Area Determination for Microporous/Mesoporous Materials: The Case of Mesoporous FAU-Y Zeolites. *Langmuir* **2018**, *34* (47), 14134–14142. https://doi.org/10.1021/acs.langmuir.8b02144.

(10) Camblor, M. A.; Corma, A.; Valencia, S. Characterization of Nanocrystalline Zeolite Beta. *Microporous and Mesoporous Materials* **1998**, *25* (1–3), 59–74. https://doi.org/10.1016/S1387-1811(98)00172-3.

(11) Goel, S.; Zones, S. I.; Iglesia, E. Encapsulation of Metal Clusters within MFI via Interzeolite Transformations and Direct Hydrothermal Syntheses and Catalytic Consequences of Their Confinement. *J. Am. Chem. Soc.* **2014**, *136* (43), 15280–15290. https://doi.org/10.1021/ja507956m.

(12) Cho, H. J.; Kim, D.; Xu, B. Pore Size Engineering Enabled Selectivity Control in Tandem Catalytic Upgrading of Cyclopentanone on Zeolite-Encapsulated Pt Nanoparticles. *ACS Catal.* **2020**, *10* (15), 8850–8859. https://doi.org/10.1021/acscatal.0c01542.

(13) Fairén-Jiménez, D.; Carrasco-Marín, F.; Moreno-Castilla, C. Adsorption of Benzene, Toluene, and Xylenes on Monolithic Carbon Aerogels from Dry Air Flows. *Langmuir* **2007**, *23* (20), 10095–10101. https://doi.org/10.1021/la701458h.

(14) Morales-Pacheco, P.; Domínguez, J. M.; Bucio, L.; Alvarez, F.; Sedran, U.; Falco, M. Synthesis of FAU(Y)- and MFI(ZSM5)-Nanosized Crystallites for Catalytic Cracking of 1,3,5-Triisopropylbenzene. *Catalysis Today* **2011**, *166* (1), 25–38. https://doi.org/10.1016/j.cattod.2010.07.005.

(15) Chupin, J.; Gnep, N. S.; Lacombe, S.; Guisnet, M. Influence of the Metal and of the Support on the Activity and Stability of Bifunctional Catalysts for Toluene Hydrogenation. *Applied Catalysis A: General* **2001**, *206* (1), 43–56. https://doi.org/10.1016/S0926-860X(00)00585-8.

(16) Lin, S. D.; Vannice, M. A. Hydrogenation of Aromatic Hydrocarbons over Supported Pt Catalysts .III. Reaction Models for Metal Surfaces and Acidic Sites on Oxide Supports. *Journal of Catalysis* **1993**, *143* (2), 563–572. https://doi.org/10.1006/jcat.1993.1299.

(17) Fischer, A. F.; Iglesia, E. The Nature of "Hydrogen Spillover": Site Proximity Effects and Gaseous Intermediates in Hydrogenation Reactions Mediated by Inhibitor-Scavenging Mechanisms. *Journal of Catalysis* **2023**, *420*, 68–88. https://doi.org/10.1016/j.jcat.2022.11.013.

(18) Gounder, R.; Iglesia, E. The Catalytic Diversity of Zeolites: Confinement and Solvation Effects within Voids of Molecular Dimensions. *Chem. Commun.* **2013**, *49* (34), 3491. https://doi.org/10.1039/c3cc40731d.

(19) Vasiurrahaman, M. The Hydrogenation of Toluene and O-, m-, and p-Xylene over Palladium II. Reaction Model. *Journal of Catalysis* **1991**, *127* (1), 267–275. https://doi.org/10.1016/0021-9517(91)90225-S.

(20) Gonzo, E.; Boudart, M. Catalytic Hydrogenation of Cyclohexene3. Gas-Phase and Liquid-Phase Reaction on Supported Palladium. *Journal of Catalysis* **1978**, *52* (3), 462–471. https://doi.org/10.1016/0021-9517(78)90352-4.

(21) Fogler, H. *Elements of Chemical Reaction Engineering*, 6th ed.; Pearson Education, Limited: Hoboken, 2020.

(22) Higgins, J. B.; LaPierre, R. B.; Schlenker, J. L.; Rohrman, A. C.; Wood, J. D.; Kerr, G. T.; Rohrbaugh, W. J. The Framework Topology of Zeolite Beta. *Zeolites* **1988**, *8* (6), 446–452. https://doi.org/10.1016/S0144-2449(88)80219-7.

(23) Arora, S. S.; Bhan, A. Kinetics of Aromatics Hydrogenation on HBEA. *Journal of Catalysis* **2020**, *383*, 24–32. https://doi.org/10.1016/j.jcat.2019.12.039.

(24) Jae, J.; Tompsett, G. A.; Foster, A. J.; Hammond, K. D.; Auerbach, S. M.; Lobo, R. F.; Huber, G. W. Investigation into the Shape Selectivity of Zeolite Catalysts for Biomass Conversion. *Journal of Catalysis* **2011**, *279* (2), 257–268. https://doi.org/10.1016/j.jcat.2011.01.019.

(25) Roque-Malherbe, R.; Wendelbo, R.; Mifsud, A.; Corma, A. Diffusion of Aromatic Hydrocarbons in H-ZSM-5, H-Beta, and H-MCM-22 Zeolites. *J. Phys. Chem.* **1995**, *99* (38), 14064–14071. https://doi.org/10.1021/j100038a043.
